# Fertility Challenges in Asthmatic Women: Examining the Complexities of Pregnancy Loss, Infertility, and Assisted Reproductive Technologies

**DOI:** 10.7759/cureus.43104

**Published:** 2023-08-07

**Authors:** Mohammed Yusuf D Shaikh, Mariam D Shaikh, Shoyeb Hirani, Aditya Nanote, Roshan Prasad, Mayur Wanjari

**Affiliations:** 1 Medicine, Jawaharlal Nehru Medical College, Datta Meghe Institute of Higher Education and Research, Wardha, IND; 2 Obstetrics and Gynecology, Dr. D. Y. Patil Vidyapeeth, Pune, IND; 3 Medicine, Mahatma Gandhi Mission (MGM) Medical College and Hospital, Aurangabad, IND; 4 Medicine and Surgery, Jawaharlal Nehru Medical College, Datta Meghe Institute of Higher Education and Research, Wardha, IND; 5 Research and Development, Jawaharlal Nehru Medical College, Datta Meghe Institute of Higher Education and Research, Wardha, IND

**Keywords:** multidisciplinary approach, medication management, in vitro fertilization, assisted reproductive technologies, infertility, pregnancy loss, fertility challenges, asthma

## Abstract

Asthma is a prevalent chronic respiratory condition affecting a significant portion of women of reproductive age. While the impact of asthma on general health and well-being has been extensively studied, its association with fertility challenges in women remains an area of growing concern. This review article explores the complexities surrounding fertility challenges in asthmatic women, specifically focusing on pregnancy loss, infertility, and the utilization of assisted reproductive technologies (ARTs). Various factors contribute to the heightened risk of pregnancy loss in asthmatic women, including the systemic inflammation associated with asthma, suboptimal asthma control, medication usage, and comorbidities. The review highlights the need for multidisciplinary management approaches to optimize asthma control before and during pregnancy, reducing the risk of adverse pregnancy outcomes. Furthermore, the review investigates the potential impact of asthma on female fertility and the underlying mechanisms involved. Asthma-related factors, such as chronic inflammation, altered hormonal balance, and medication effects, may disrupt the delicate reproductive processes, leading to infertility. It emphasizes the importance of comprehensive fertility evaluations and personalized treatment strategies for asthmatic women experiencing difficulties conceiving. Additionally, the article explores the utilization of ARTs, including in vitro fertilization (IVF) and intracytoplasmic sperm injection (ICSI), in asthmatic women. It discusses the safety considerations and potential challenges associated with these techniques, such as the impact of asthma medications on oocyte quality, the effects of hormonal stimulation on asthma control, and the risk of exacerbations during the IVF process. The review underscores the importance of collaborative efforts among healthcare providers, including allergists, pulmonologists, obstetricians, and fertility specialists, to ensure optimal management of asthmatic women seeking to conceive. It emphasizes the significance of preconception counseling, meticulous asthma control, appropriate medication management, and individualized fertility treatments to enhance the reproductive outcomes in this population.

## Introduction and background

Asthma is a chronic respiratory condition characterized by inflammation and narrowing of the airways, leading to symptoms such as wheezing, shortness of breath, coughing, and chest tightness. According to the World Health Organization (WHO), asthma affects approximately 339 million people worldwide, making it a significant public health concern. The prevalence of asthma varies across different populations and is influenced by various factors such as genetic predisposition, environmental triggers, and lifestyle factors [1,2].

While the impact of asthma on lung function and overall health has been extensively studied, its effects on female reproductive health, specifically fertility and pregnancy outcomes, have gained increasing attention in recent years. Asthmatic women may face unique challenges when conceiving, carrying a pregnancy to term, and undergoing assisted reproductive technologies (ARTs). Understanding the complexities surrounding fertility issues in asthmatic women is crucial for healthcare professionals to provide appropriate care and support [3,4].

This review article examines the fertility challenges faced by asthmatic women, focusing on the complexities of pregnancy loss, infertility, and the use of ARTs. By synthesizing existing literature and research findings, this article aims to provide a comprehensive overview of the topic, highlighting the potential mechanisms underlying these fertility challenges and exploring the impact of asthma medications on reproductive outcomes. Additionally, this review article seeks to raise awareness among healthcare professionals about the need for a multidisciplinary approach in managing asthmatic women planning for pregnancy. This article aims to advance clinical practice and improve reproductive health outcomes for asthmatic women by addressing the gaps in knowledge and identifying areas for future research.

## Review

Asthma and its impact on female reproductive health

Overview of Asthma and Its Symptoms

Asthma is a chronic inflammatory condition of the airways characterized by recurrent wheezing, shortness of breath, coughing, and chest tightness. It affects individuals of all ages and can significantly affect female reproductive health [2]. In asthma, the airways become inflamed, leading to increased sensitivity and narrowing of the air passages. This inflammation is typically triggered by exposure to certain substances or environmental factors, such as allergens, pollutants, or respiratory infections. The resulting constriction of the airways and excess mucus production can cause symptoms that range from mild to severe, and the frequency and severity of these symptoms may vary among individuals with asthma [2].

Common asthma symptoms include wheezing and a high-pitched whistling sound during breathing, especially during expiration. Shortness of breath, characterized by a feeling of breathlessness or difficulty taking full breaths, is another hallmark symptom. Coughing is often present, particularly at night or in response to triggers. Chest tightness or discomfort may also be experienced, and individuals with asthma may describe a sensation of pressure or squeeze in the chest [5].

Link Between Asthma and Hormonal Imbalances

Research suggests that asthma and hormonal imbalances in women may be linked. Hormones are vital in regulating the menstrual cycle and ensuring normal reproductive function. Disturbances in hormonal levels can have a significant impact on fertility outcomes. In the context of asthma, the chronic inflammation and immune dysregulation associated with the condition can disrupt the delicate hormonal balance. Asthma-related inflammation can potentially affect the hypothalamic-pituitary-ovarian axis, which regulates hormone production and release. This disruption in hormonal balance may have implications for ovulation, follicular development, and overall reproductive health in women with asthma. Furthermore, the immune dysregulation seen in asthma may contribute to further disturbances in hormone levels, potentially influencing fertility outcomes. However, more research is needed to fully understand the precise mechanisms and the extent of the link between asthma and hormonal imbalances in women. Nonetheless, recognizing and addressing these potential connections is crucial for providing appropriate care to asthmatic women seeking to conceive [6-11].

Effects of Asthma on the Menstrual Cycle and Ovulation

The effects of asthma on the menstrual cycle and ovulation have been of interest in understanding the impact of asthma on female reproductive health. Several studies have reported associations between asthma and menstrual irregularities. Women with asthma are more likely to experience irregular menstrual cycles characterized by variations in cycle length, missed periods, or unpredictable bleeding patterns. Also, asthmatic women may have longer menstrual durations than non-asthmatic women [12].

Furthermore, emerging evidence suggests that asthma may influence ovulation, a critical fertility process. Ovulation is the release of a mature egg from the ovary, allowing for the possibility of fertilization. Disruptions in ovulation can significantly impact fertility potential. Some studies indicate that asthma may affect ovulation by disrupting hormonal regulation, specifically the balance of estrogen and progesterone, which are essential for proper ovulatory function [13].

Decreased fertility potential has also been observed in asthmatic women. Oligomenorrhea, a condition characterized by infrequent menstruation, has been reported to be more prevalent in women with asthma. This infrequent ovulation can hinder the chances of conceiving. However, it is important to note that the relationship between asthma and fertility is complex, and additional factors such as asthma severity, control, and comorbidities may further influence fertility outcomes [14].

The specific mechanisms underlying the effects of asthma on the menstrual cycle and ovulation are not yet fully understood. However, chronic inflammation and immune dysregulation associated with asthma may contribute to hormonal imbalances and disrupt the normal functioning of the reproductive system. These disturbances can ultimately impact asthmatic women's menstrual regularity, ovulation, and fertility potential [8].

Impact of Asthma Medications on Fertility

The impact of asthma medications on fertility in women remains an area of active research and discussion. Certain asthma medications, such as corticosteroids, are commonly prescribed to manage asthma symptoms and reduce inflammation. However, concerns have been raised about the potential effects of these medications on fertility and reproductive outcomes. While corticosteroids are generally considered safe during pregnancy, their long-term effects on fertility and ART outcomes require further investigation [15]. Healthcare providers need to consider the potential effects of asthma and its medications on female reproductive health when managing asthmatic women who are planning for pregnancy. Close collaboration between asthma specialists and fertility experts can help optimize asthma control while minimizing potential fertility and reproductive outcomes risks. Further research is needed to elucidate the underlying mechanisms and develop evidence-based guidelines for managing asthmatic women seeking to conceive.

Pregnancy loss in asthmatic women

Overview of Pregnancy Loss and Its Types

Pregnancy loss refers to the spontaneous termination of a pregnancy before the fetus reaches a viable stage. It can occur at various stages, including early pregnancy loss (miscarriage) and late pregnancy loss (stillbirth). Miscarriage is typically defined as losing a pregnancy before 20 weeks of gestation, while stillbirth refers to fetal demise after 20 weeks [16].

Research Studies on the Association Between Asthma and Pregnancy Loss

Research studies investigating the association between asthma and pregnancy loss have provided valuable insights into this complex relationship. Although the evidence is not yet definitive, several studies have indicated a potential increased risk of pregnancy loss in women with asthma compared to those without asthma. These findings suggest that asthma may contribute to adverse pregnancy outcomes. However, it is important to note that conflicting results also exist, and the relationship between asthma and pregnancy loss is influenced by various factors [17,18-24].

One important factor is the severity of asthma. Studies have suggested that women with more severe asthma may be at a higher risk of experiencing pregnancy loss. Poorly controlled asthma, characterized by frequent exacerbations and inadequate management, may contribute to increased systemic inflammation and oxidative stress, potentially impacting pregnancy outcomes. On the other hand, women with well-controlled asthma may have a lower risk of pregnancy loss than those with uncontrolled asthma [25].

Asthma control is another crucial factor influencing the association between asthma and pregnancy loss. Studies have shown that women with better asthma control have a reduced risk of adverse pregnancy outcomes, including loss. Achieving optimal asthma control through appropriate management strategies, including medication adherence, regular monitoring, and avoidance of triggers, may help mitigate the potential risks to pregnancy [26].

Comorbidities, such as allergies, obesity, and other respiratory conditions, can also influence the association between asthma and pregnancy loss. These factors may interact with asthma and contribute to adverse pregnancy outcomes. Understanding and considering the presence of these comorbidities are important when examining the relationship between asthma and pregnancy loss [27].

Potential Mechanisms Linking Asthma and Pregnancy Loss

The mechanisms linking asthma and pregnancy loss are not fully understood, but several potential pathways have been proposed. One potential mechanism is the chronic inflammation and immune dysregulation characteristic of asthma. These factors can contribute to increased oxidative stress, impairing placental function and leading to pregnancy complications and loss. Asthma-related changes in hormone levels, such as alterations in progesterone and estrogen, could also play a role in pregnancy loss. The altered hormonal environment may affect the implantation of the embryo and the maintenance of pregnancy. Furthermore, systemic inflammation associated with asthma could harm the developing fetus and the placenta, increasing the risk of pregnancy loss. The complex interplay between these factors, including chronic inflammation, hormonal imbalances, and altered uterine environment, contributes to the increased susceptibility to pregnancy loss in asthmatic women. However, further research is needed to fully elucidate the specific mechanisms involved and their relative contributions to pregnancy loss in this population [28-30].

Impact of Asthma Medications on Pregnancy Loss Risk

The impact of asthma medications on the risk of pregnancy loss remains an area of investigation. Generally, most asthma medications, including short-acting bronchodilators and inhaled corticosteroids, are considered safe during pregnancy. However, the safety of systemic corticosteroids, such as oral or intravenous administration, especially in high doses, is still debatable. Some studies have suggested a potential association between oral corticosteroid use and an increased risk of pregnancy complications, including loss. Healthcare providers must weigh the benefits of asthma control against potential risks when considering medication options for asthmatic women during pregnancy [31]. Further research is needed to understand better the complex interplay between asthma and pregnancy loss, including the specific mechanisms involved and the potential impact of asthma medications. This knowledge will help guide clinical management strategies and optimize outcomes for asthmatic women planning for pregnancy.

Infertility in asthmatic women

Definition and Causes of Infertility

Infertility is the inability to conceive after one year of regular unprotected intercourse. Various factors, including ovulatory disorders, fallopian tube abnormalities, uterine abnormalities, endocrine disorders, sperm-related issues, and unexplained factors, can cause it. Understanding the specific causes of infertility is essential for effective management and treatment [32].

Studies Investigating the Relationship Between Asthma and Infertility

The studies have examined the potential association between asthma and infertility in women, shedding light on the complex relationship between these two conditions. While some research suggests a higher prevalence of infertility among women with asthma than those without asthma, the evidence remains limited and contradictory, with other studies finding no significant association [33].

Factors such as asthma severity, control, and comorbidities are likely to influence the impact of asthma on female fertility. Women with poorly controlled asthma or more severe forms of the condition may experience a higher risk of infertility. Additionally, comorbidities, such as hormonal imbalances or other reproductive disorders, could contribute to the association between asthma and infertility [14]. It is important to note that the mechanisms underlying this relationship are not yet fully understood. Further research is necessary to elucidate the specific pathways through which asthma may affect fertility, including the potential role of chronic inflammation, hormonal disruptions, or the impact of asthma medications. Additionally, large-scale prospective studies are needed to provide more robust evidence and clarify the potential confounding factors that may influence the association between asthma and infertility.

Impact of Asthma Severity and Control on Fertility

The severity and control of asthma can potentially affect fertility in women. Poorly controlled asthma, characterized by frequent exacerbations, may lead to increased systemic inflammation and oxidative stress, adversely affecting reproductive function. Additionally, asthma-related hormonal imbalances and the use of oral corticosteroids for asthma management, particularly in high doses, may further impact fertility outcomes. On the other hand, achieving good asthma control through appropriate management and treatment strategies may help optimize fertility potential [12].

Effect of Asthma Medications on Fertility Treatments

Asthma medications, particularly corticosteroids, are commonly used to manage asthma symptoms and reduce airway inflammation. Concerns have been raised regarding the potential effects of these medications on fertility treatments, such as ARTs. While the available evidence is limited, studies generally suggest that inhaled corticosteroids, which are the mainstay of asthma treatment, do not adversely affect fertility or the success of ART. However, further research is needed to investigate the potential impact of systemic corticosteroids on fertility treatments and to develop evidence-based guidelines for managing asthmatic women undergoing fertility interventions [34].

A comprehensive approach that addresses asthma control and fertility factors is crucial in managing infertility in asthmatic women. Collaboration between asthma specialists and fertility experts can help optimize asthma management while minimizing potential risks to fertility. Future research should focus on elucidating the mechanisms underlying the relationship between asthma and infertility and establishing guidelines for managing asthmatic women undergoing fertility treatments [35].

ARTs and asthmatic women

Introduction to ART and Its Various Techniques Such As In Vitro Fertilization and Intrauterine Insemination

ARTs are advanced fertility treatments that assist individuals or couples in achieving pregnancy. ART encompasses various techniques, including in vitro fertilization (IVF), intrauterine insemination (IUI), intracytoplasmic sperm injection (ICSI), and others. These techniques involve manipulating eggs, sperm, and embryos outside the body to facilitate fertilization and implantation [36].

Considerations for Asthmatic Women Undergoing ART

Asthmatic women undergoing ART may require additional considerations to optimize their reproductive outcomes. Assessing asthma control and optimizing asthma management before proceeding with ART procedures is important. Asthma control is crucial to minimize potential complications and ensure a healthy pregnancy. Close communication and collaboration between asthma specialists and fertility experts are essential to develop an individualized treatment plan that addresses asthma management and fertility goals [37].

Safety of Asthma Medications During ART Procedures

The safety of asthma medications during ART procedures is a key concern for asthmatic women. Most asthma medications, such as inhaled corticosteroids and short-acting bronchodilators, are considered safe for use during ART. However, the specific medication regimen may need to be adjusted based on individual needs and pregnancy plans. It is important for healthcare providers to carefully evaluate the risks and benefits of different asthma medications and consider any potential impact on fertility treatments. Close monitoring and regular communication between the asthma specialist and the fertility team are necessary to ensure optimal asthma control while minimizing any risks to the ART process [38].

Effectiveness of ART in Achieving Successful Pregnancies in Asthmatic Women

Studies have shown that the success rates of ART in achieving successful pregnancies are generally similar between asthmatic and non-asthmatic women. Asthma itself does not affect ART procedures' overall success significantly. However, asthma severity, control, and the presence of other underlying fertility factors may influence the outcomes. Optimizing asthma management and controlling other relevant factors are important steps to maximize the chances of a successful pregnancy through ART [4,33,39].

Management and strategies for asthmatic women planning pregnancy

Preconception Counseling and Asthma Management

Preconception counseling is vital in optimizing reproductive outcomes for asthmatic women planning for pregnancy. During preconception counseling, healthcare providers should assess asthma severity, control, and medication use. Asthmatic women should be educated about the potential impact of asthma on fertility and pregnancy and the importance of asthma management. They should be encouraged to achieve optimal asthma control before conceiving to minimize potential risks to both mother and baby [40].

Importance of Asthma Control During Pregnancy

Maintaining good asthma control during pregnancy is crucial for the health and well-being of both the mother and the developing fetus. Poorly controlled asthma during pregnancy increases the risk of pregnancy complications, such as preterm birth, low birth weight, preeclampsia, and exacerbation of asthma symptoms. Asthmatic women should be closely monitored throughout pregnancy, and treatment adjustments should be made to ensure optimal asthma control [41].

Adjusting Asthma Medications and Treatment Plans

Asthmatic women planning for pregnancy may need adjustments to their asthma medications and treatment plans. Some medications, such as inhaled corticosteroids, are considered safe during pregnancy and should be continued to maintain asthma control. However, specific medication regimens may need to be individualized based on the severity of asthma and the patient's specific needs. Due to potential risks, oral corticosteroids should be minimized, especially during pregnancy. Close collaboration between the asthma specialist and the obstetrician is essential to develop an appropriate treatment plan that balances asthma control with minimizing potential risks to the developing fetus [42].

Collaborative Care Between Asthma and Fertility Specialists

Collaborative care between asthma specialists and fertility experts is vital to ensure comprehensive and personalized management for asthmatic women planning pregnancy. A multidisciplinary approach allows for a thorough assessment of asthma control, optimization of medication regimens, and consideration of potential interactions between asthma and fertility treatments. Open communication between the healthcare providers involved in the care of asthmatic women can help address any concerns, provide appropriate guidance, and ensure coordinated and cohesive management throughout the journey from planning to pregnancy [43].

Future directions and research needs

Current Gaps in Knowledge and Research

Limited understanding of the underlying mechanisms: While the association between asthma and fertility challenges has been established, the biological mechanisms linking asthma to pregnancy loss and infertility remain poorly understood. Further research is necessary to elucidate these mechanisms and identify potential therapeutic targets. Investigating the impact of chronic inflammation, immune dysregulation, hormonal imbalances, and other asthma-related factors on fertility outcomes is crucial for a comprehensive understanding of this complex relationship [22].

Inconsistencies in study findings: Studies examining the association between asthma and fertility outcomes have yielded inconsistent findings, possibly due to variations in study designs, sample sizes, and participant characteristics. These discrepancies hinder the establishment of robust and reliable evidence. More high-quality studies with standardized methodologies, larger sample sizes, and diverse populations are needed to clarify the relationship between asthma and fertility challenges, providing a more comprehensive understanding of the topic [44].

Safety and efficacy of asthma medications: The safety and efficacy of asthma medications during pregnancy and fertility treatments require further investigation. Specifically, the long-term effects of asthma medications, including corticosteroids, on fertility outcomes and the success of ARTs must be clarified. Research must assess the potential risks associated with different asthma medications, optimal medication regimens during pregnancy and fertility treatments, and their impact on reproductive health outcomes [45].

Areas for Future Investigation and Studies

Mechanistic studies: In-depth studies are needed to unravel the underlying biological pathways and immune mechanisms contributing to fertility challenges in asthmatic women. By understanding these mechanisms, researchers can identify specific targets for intervention and develop management strategies tailored to the unique needs of asthmatic women. Exploring the interplay between asthma-related inflammation, hormonal imbalances, and oxidative stress can provide valuable insights into the complex relationship between asthma and fertility outcomes [46].

Large-scale prospective studies: Conducting large-scale prospective studies with standardized methodologies is essential to provide more reliable and consistent evidence on the association between asthma and fertility outcomes. These studies should consider factors such as asthma severity, control, comorbidities, and the impact of specific asthma medications on reproductive health. By enrolling diverse populations of asthmatic women and carefully controlling for confounding factors, researchers can obtain more robust evidence on the impact of asthma on fertility, pregnancy loss, and the success of fertility treatments [39].

Long-term follow-up studies: Long-term follow-up studies that track the reproductive outcomes of asthmatic women over time can provide valuable insights into the long-term effects of asthma on fertility, pregnancy, and child health. These studies can assess the impact of various factors, including asthma control, medication use, and other comorbidities, on reproductive health outcomes. By observing the long-term implications of asthma on fertility, researchers can identify potential areas for intervention and develop targeted strategies to optimize reproductive health outcomes for asthmatic women [47].

Importance of Multidisciplinary Collaboration

Develop comprehensive guidelines: Multidisciplinary collaboration can lead to developing evidence-based guidelines for managing asthmatic women planning for pregnancy. By pooling the expertise of different disciplines, these guidelines can provide healthcare providers with standardized approaches to optimize asthma control and reproductive health outcomes. The consensus among specialists ensures that recommendations consider asthma management and fertility goals, promoting the best care for asthmatic women seeking to conceive [48].

Improve patient care: Collaborative care allows for a holistic approach that addresses asthma management and fertility goals. By fostering open communication and coordination between healthcare providers, asthmatic women can receive personalized care that considers their unique needs and optimizes their chances of a successful pregnancy. This multidisciplinary approach ensures that asthma control is prioritized. At the same time, fertility treatment plans are designed and implemented to minimize potential risks to the mother and the developing fetus [49].

Facilitate research and knowledge exchange: Collaboration between different disciplines enables the exchange of knowledge, expertise, and research findings. Asthma specialists, fertility experts, obstetricians, and researchers can share their unique insights and experiences, accelerating research progress and promoting innovation in the field. This collaborative effort also facilitates the translation of research findings into clinical practice, ensuring that new knowledge is applied to improve the management and care provided to asthmatic women planning for pregnancy [50].

## Conclusions

In conclusion, fertility challenges in asthmatic women are complex and require careful consideration. Asthma can potentially impact female reproductive health, including fertility outcomes, pregnancy loss, and the use of ARTs. Factors such as asthma severity, control, and comorbidities influence the association between asthma and fertility challenges. Although not fully understood, the underlying mechanisms linking asthma to fertility challenges may involve chronic inflammation, immune dysregulation, hormonal imbalances, and the effects of asthma medications. Clinical practice should emphasize preconception counseling, optimizing asthma management, and adjusting medication regimens to balance asthma control and potential risks to fertility and pregnancy outcomes. Collaborative care among asthma specialists, fertility experts, and obstetricians is crucial for providing comprehensive and coordinated care. Future advancements should focus on further research to elucidate the underlying mechanisms, conduct large-scale prospective studies, and foster multidisciplinary collaboration to develop comprehensive guidelines. By addressing these areas, we can enhance the management and care provided to asthmatic women planning for pregnancy, leading to improved reproductive health outcomes and the well-being of both mother and child.

## References

[1] Trivedi M, Denton E (2019). Asthma in children and adults—what are the differences and what can they tell us about asthma?. Front Pediatr.

[2] (2023). Asthma. https://www.who.int/news-room/fact-sheets/detail/asthma.

[3] Bravo-Solarte DC, Garcia-Guaqueta DP, Chiarella SE (2023). Asthma in pregnancy. Allergy Asthma Proc.

[4] Juul Gade E, Thomsen SF, Lindenberg S, Backer V (2014). Female asthma has a negative effect on fertility: what is the connection?. ISRN Allergy.

[5] Gong H (1990). Wheezing and Asthma. Clinical Methods: The History, Physical, and Laboratory Examinations.

[6] Zein JG, Erzurum SC (2015). Asthma is different in women. Curr Allergy Asthma Rep.

[7] Yung JA, Fuseini H, Newcomb DC (2018). Hormones, sex, and asthma. Ann Allergy Asthma Immunol.

[8] Calcaterra V, Nappi RE, Farolfi A, Tiranini L, Rossi V, Regalbuto C, Zuccotti G (2022). Perimenstrual asthma in adolescents: a shared condition in pediatric and gynecological endocrinology. Children (Basel).

[9] Baldaçara RP, Silva I (2017). Association between asthma and female sex hormones. Sao Paulo Med J.

[10] Tam A, Morrish D, Wadsworth S, Dorscheid D, Man SF, Sin DD (2011). The role of female hormones on lung function in chronic lung diseases. BMC Womens Health.

[11] Lam GY, Goodwin J, Wilcox PG, Quon BS (2021). Sex disparities in cystic fibrosis: review on the effect of female sex hormones on lung pathophysiology and outcomes. ERJ Open Res.

[12] Xu Y, Zhou ZY, Pan JX, Huang HF (2022). Associations between asthma and polycystic ovary syndrome: current perspectives. Front Endocrinol (Lausanne).

[13] Bláfoss J, Hansen AV, Lauesgaard SSM, Ali Z, Ulrik CS (2019). Female asthma and atopy - impact on fertility: a systematic review. J Asthma Allergy.

[14] Guarnieri G, Iervolino M, Cavallone S, Unfer V, Vianello A (2023). The “Asthma-Polycystic Ovary Overlap Syndrome” and the therapeutic role of Myo-inositol. Int J Mol Sci.

[15] Crowe HM, Wise LA, Wesselink AK (2020). Association of asthma diagnosis and medication use with fecundability: a prospective cohort study. Clin Epidemiol.

[16] Dugas C, Slane VH (2023). Miscarriage. https://www.ncbi.nlm.nih.gov/books/NBK532992/.

[17] Wang H, Li N, Huang H (2020). Asthma in pregnancy: pathophysiology, diagnosis, whole-course management, and medication safety. Can Respir J.

[18] Tidemandsen C, Egerup P, Ulrik CS (2022). Asthma is associated with pregnancy loss and recurrent pregnancy loss: a nationwide cohort study. J Allergy Clin Immunol Pract.

[19] Tidemandsen C, Egerup P, Ulrik CS (2021). Recurrent pregnancy loss and asthma: a nationwide study. Eur Respir J.

[20] Magnus MC, Karlstad Ø, Parr CL (2019). Maternal history of miscarriages and measures of fertility in relation to childhood asthma. Thorax.

[21] Al-Hussainy A, Mohammed R (2021). Consequences of maternal psychological stress during pregnancy for the risk of asthma in the offspring. Scand J Immunol.

[22] Jöud A, Nilsson-Condori E, Schmidt L, Ziebe S, Vassard D, Mattsson K (2022). Infertility, pregnancy loss and assisted reproduction in women with asthma: a population-based cohort study. Hum Reprod.

[23] Fazel N, Kundi M, Jensen-Jarolim E (2018). Prospective cohort study of pregnancy complications and birth outcomes in women with asthma. Arch Gynecol Obstet.

[24] Huang S, Hee JY, Zhang YO, Gongye R, Zou S, Tang K (2022). Association between pregnancy and pregnancy loss with COPD in Chinese women: the China Kadoorie Biobank study. Front Public Health.

[25] Kostakou E, Kaniaris E, Filiou E (2019). Acute severe asthma in adolescent and adult patients: current perspectives on assessment and management. J Clin Med.

[26] Bonham CA, Patterson KC, Strek ME (2018). Asthma outcomes and management during pregnancy. Chest.

[27] Kaplan A, Szefler SJ, Halpin DM (2020). Impact of comorbid conditions on asthmatic adults and children. NPJ Prim Care Respir Med.

[28] Lebold KM, Jacoby DB, Drake MG (2020). Inflammatory mechanisms linking maternal and childhood asthma. J Leukoc Biol.

[29] Douros K, Moustaki M, Tsabouri S, Papadopoulou A, Papadopoulos M, Priftis KN (2017). Prenatal maternal stress and the risk of asthma in children. Front Pediatr.

[30] Cevhertas L, Ogulur I, Maurer DJ (2020). Advances and recent developments in asthma in 2020. Allergy.

[31] Gregersen TL, Ulrik CS (2013). Safety of bronchodilators and corticosteroids for asthma during pregnancy: what we know and what we need to do better. J Asthma Allergy.

[32] (2023). Infertility. https://www.who.int/news-room/fact-sheets/detail/infertility.

[33] Gade EJ, Thomsen SF, Lindenberg S, Backer V (2016). Fertility outcomes in asthma: a clinical study of 245 women with unexplained infertility. Eur Respir J.

[34] Alangari AA (2014). Corticosteroids in the treatment of acute asthma. Ann Thorac Med.

[35] Gade EJ, Tidemandsen C, Hansen AV, Ulrik CS, Backer V (2022). Challenges in the successful management of asthma during conception, pregnancy and delivery. Breathe (Sheff).

[36] Jain M, Singh M (2023). Assisted Reproductive Technology (ART) Techniques. https://www.ncbi.nlm.nih.gov/books/NBK576409/.

[37] Murphy VE (2015). Managing asthma in pregnancy. Breathe (Sheff).

[38] Sharma S, Hashmi MF, Chakraborty RK (2023). Asthma Medications. https://www.ncbi.nlm.nih.gov/books/NBK531455/.

[39] Grosso A, Locatelli F, Gini E, Albicini F, Tirelli C, Cerveri I, Corsico AG (2018). The course of asthma during pregnancy in a recent, multicase-control study on respiratory health. Allergy Asthma Clin Immunol.

[40] Farahi N, Zolotor A (2013). Recommendations for preconception counseling and care. Am Fam Physician.

[41] Bakhireva LN, Schatz M, Jones KL, Chambers CD (2008). Organization of teratology information specialists collaborative research group: asthma control during pregnancy and the risk of preterm delivery or impaired fetal growth. Ann Allergy Asthma Immunol Off Publ Am Coll Allergy Asthma Immunol.

[42] Chambers CD, Krishnan JA, Alba L (2021). The safety of asthma medications during pregnancy and lactation: clinical management and research priorities. J Allergy Clin Immunol.

[43] Lim A, Stewart K, Abramson MJ, Walker SP, George J (2012). Multidisciplinary approach to management of maternal asthma (MAMMA [copyright]): the PROTOCOL for a randomized controlled trial. BMC Public Health.

[44] Veroniki AA, Tsokani S, White IR (2021). Prevalence of evidence of inconsistency and its association with network structural characteristics in 201 published networks of interventions. BMC Med Res Methodol.

[45] (2023). Steroid Inhalers/Pills for Asthma Linked to Heightened Risk of Brittle Bones and Fractures. https://www.bmj.com/company/newsroom/steroid-inhalers-pills-for-asthma-linked-to-heightened-risk-of-brittle-bones-and-fractures/.

[46] Laubhahn K, Phelan KJ, Jackson DJ, Altman MC, Schaub B (2023). What have mechanistic studies taught us about childhood asthma?. J Allergy Clin Immunol Pract.

[47] Tidemandsen C, Vejen Hansen A, Backer V, Gade EJ, Ali Z, Suppli Ulrik C (2020). Fertility treatment resulting in live births in women with asthma - associated with perennial allergy?. J Asthma Allergy.

[48] Bain E, Pierides KL, Clifton VL, Hodyl NA, Stark MJ, Crowther CA, Middleton P (2014). Interventions for managing asthma in pregnancy. Cochrane Database Syst Rev.

[49] Qamar N, Pappalardo AA, Arora VM, Press VG (2011). Patient-centered care and its effect on outcomes in the treatment of asthma. Patient Relat Outcome Meas.

[50] Levy BD, Noel PJ, Freemer MM (2015). Future research directions in asthma: an NHLBI Working Group report. Am J Respir Crit Care Med.

